# Supplementary material for: The Construction and Exploration of a Comprehensive MicroRNA Centered Regulatory Network in Foxtail Millet (Setaria italica L.)
Source: Front Plant Sci. 2022 May 6;13:848474. doi: 10.3389/fpls.2022.848474 (PMC9121102; doi:10.3389/fpls.2022.848474)
Supplement: Supplementary file 6 [file Data_Sheet_6.zip › Sit-miR395j_Seita.9G409100.1_217_TPlot.pdf]

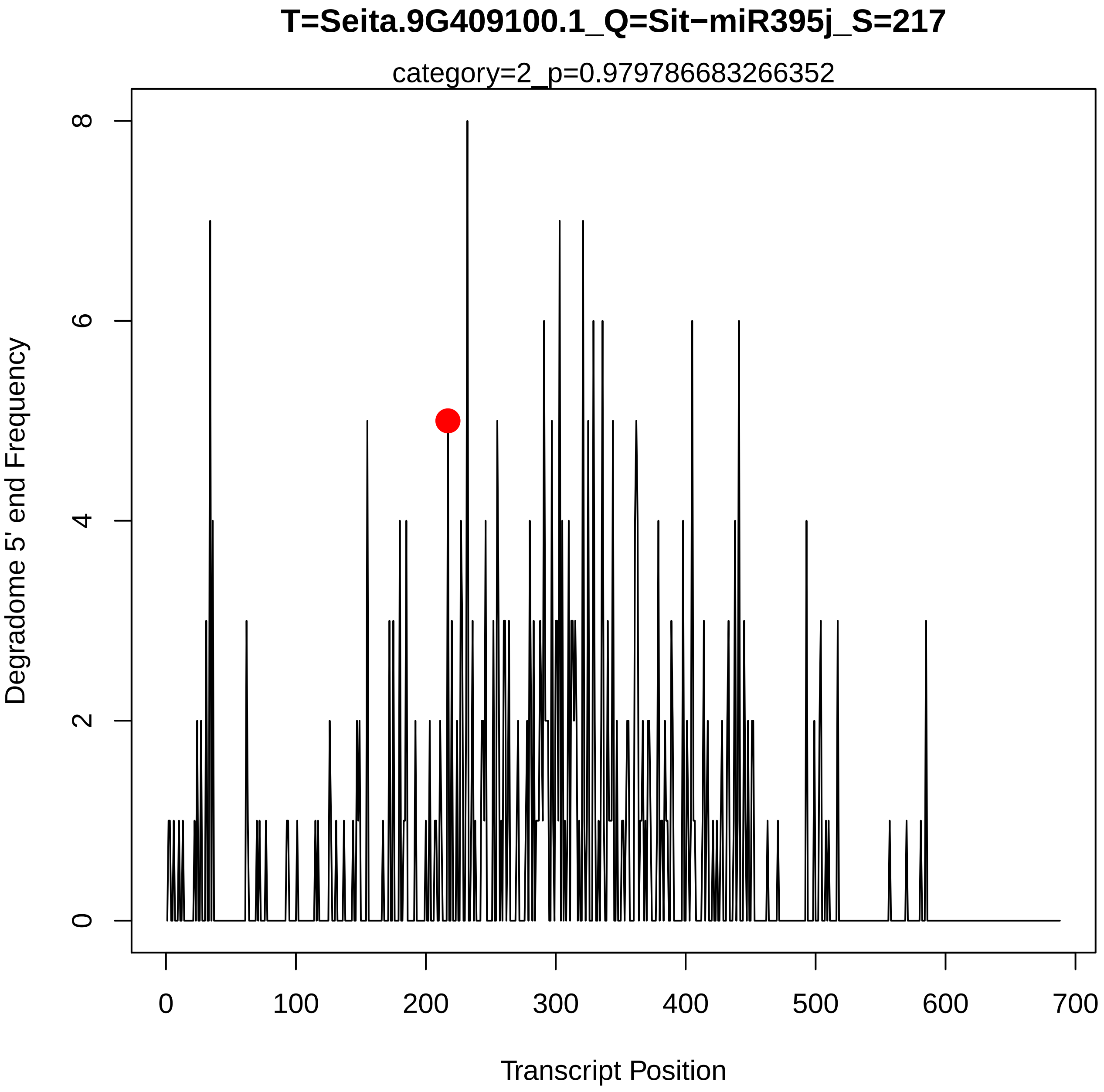
T=Seita.9G409100.1_Q=Sit−miR395j_S=217
category=2_p=0.979786683266352
Degradome 5' end Frequency
0
2
4
6
8
0
100
200
300
400
500
600
700
Transcript Position
